# Imported endemic mycoses in Spain: Evolution of hospitalized cases, clinical characteristics and correlation with migratory movements, 1997-2014

**DOI:** 10.1371/journal.pntd.0006245

**Published:** 2018-02-15

**Authors:** Daniel Molina-Morant, Adrián Sánchez-Montalvá, Fernando Salvador, Augusto Sao-Avilés, Israel Molina

**Affiliations:** Tropical Medicine Unit, Infectious Diseases Department. PROSICS Barcelona (International Health Program of the Catalan Health Institute), Hospital Universitari Vall d’Hebron, Universitat Autònoma de Barcelona, Barcelona, Spain; Universidad de Antioquia, COLOMBIA

## Abstract

Endemic mycoses are systemic fungal infections. Histoplasmosis is endemic in all temperate areas of the world; coccidioidomycosis and paracoccidioidomycosis are only present in the American continent. These pathogens are not present in Spain, but in the last years there has been an increase of reported cases due to migration and temporary movements. We obtained from the Spanish hospitals records clinical and demographic data of all hospitalized cases between 1997 and 2014. There were 286 cases of histoplasmosis, 94 of Coccidioidomycosis and 25 of paracoccidioidomycosis. Overall, histoplasmosis was strongly related to HIV infection, as well as with greater morbidity and mortality. For the other mycoses, we did not find any immunosuppressive condition in most of the cases. Although we were not able to obtain data about clinical presentation of all the cases, the most frequently found was pulmonary involvement. We also found a temporal correlation between the Spanish population born in endemic countries and the number of hospitalized cases along this period. This study reflects the importance of imported diseases in non-endemic countries due to migratory movements.

## Author summary

Histoplasmosis, coccidioidomycosis and paracoccidioidomycosis are infections produced by environmental fungi. Despite the fact that these pathogens are not found in Spain, these diseases are an important health problem in many countries of the world, especially in Latin America. Due to the increased migration from these countries in Spain in the last years, we have observed a rise in the number of new reported cases of these diseases. Nevertheless, they probably remain underestimated since it is likely that physicians do not initially suspect them, and their notification is not mandatory. People who travel to endemic countries are at risk to become infected as well. In this article we used data from the Spanish hospitals records with the aim to describe the main clinical and epidemiological characteristics of these endemic mycoses in Spain, and try to find a correlation between the growing Spanish population at risk and the number of cases hospitalized. This study contributes to have a more accurate knowledge about the burden of these diseases in Spain.

## Introduction

Endemic mycoses are systemic fungal infections caused by pathogens that are endemic in temperate areas of the world. These infections are classified according to the infectivity of the fungus into two groups: produced by primary pathogens and by opportunistic pathogens [1]. The genus *Histoplasma* ssp, *Coccidioides* ssp and *Paracoccidioides* ssp belong to the first group [2].

These fungi are found in the environment as filamentous form. Infection in humans usually occurs through inhalation of microconidia that are present in contaminated bird and bat droppings. Hence, the respiratory tract is generally affected. In immunocompetent hosts the infection is usually limited and its clinical picture ranges from asymptomatic or a flu-like syndrome to an acute pneumonia, and it results in partial protection from future infection [3]. However, in patients with cellular immunodeficiency, disseminated disease can occur, with a poorer prognosis and higher mortality [4]. Due to the mechanism of transmission, they have been historically associated with certain professional occupations such as farmers, breeders, miners, biologists and cavers.

The worldwide most often described endemic mycosis is histoplasmosis, caused by *Histoplasma capsulatum* species. *Histoplasma capsulatum var*. *capsulatum* is endemic in temperate areas worldwide. In the American continent is present in the Mississippi, Ohio, and St. Lawrence River valleys, the Caribbean, southern Mexico, and certain tropical areas of Central and South America. Other areas include West and Central Africa, Zimbabwe and South Africa, Southeast Asia, and some areas of India, China and Australia [5]. In addition, in the Equatorial Africa area coexists with other pathogenic species for humans, *Histoplasma capsulatum var*. *duboisii* [6]. Coccidioidomycosis is caused by the genus *Coccidioides (C*. *immitis and C*. *posadasii*), and it is endemic in areas that include southern Arizona, the southern and central valleys of California, southwestern New Mexico, west Texas in the United States, and some parts of Mexico and Central and South America [7]. Paracoccidioidomycosis (also called South American blastomycosis), is mostly caused by *Paracoccidioides brasiliensis* and in fewer cases by *Paracoccidioides lutzii*, that are endemic in certain regions of Central and South America, especially in Brazil, where approximately 80 percent of the world cases have been reported [8].

In the recent years, especially since the beginning of the last century, there has been an increase in the number of reported cases of these infections in non endemic countries. Spain has historically received immigration from Latin America due to its cultural and historical ties, however data from endemic mycoses in Spain are scarce [9–12]. The aim of this study is to describe the evolution of hospitalized cases of histoplasmosis, coccidioidomycosis and paracoccidioidomycosis in Spain in the period between 1997 and 2014, and establish the possible correlation with demographic changes due to migration and international movements. Other objectives are to describe the clinical picture and sociodemographic characteristics of these diseases.

## Methods

### Data analysis

We carried out a retrospective study from January 1st 1997 to December 31st 2014. Data from patients admitted to Spanish hospitals and diagnosed of histoplasmosis, coccidioidomycosis or paracoccidioidomycosis were obtained from the Basic Minimum Data Set (CMBD by its acronym in Spanish), provided by Epidemiological Surveillance Section of Ministry of Health, Social Services and Equality of Spain (MSSSI by its acronym in Spanish) [13]. The CMBD classifies the information according to the International Classification of Diseases, Ninth Revision, Clinical Modification (ICD-9 CM codes 114.0 to 116.1) [14]. Most of the data is collected from the Spanish National Health System that provides free medical care to 99.5% of the Spanish population, but the CMBD has also had a gradual data coverage from private hospitals since 2005. Spain has a population of 46,468,102 inhabitants with an area of 504,645 km^2^, and it consists of 19 political regions (17 autonomous communities and 2 autonomous cities in northern Africa). The health system consists of a total of 791 public and private hospitals [15].

For each case, we collected clinical data (admission and discharge date, length of hospitalization, readmission, outcome, principal and secondary diagnoses, immunosuppressant status, clinical picture of disease and comorbidities) and socio-demographic characteristics (sex, age and autonomous community of residency). We could not obtain the nationality of the patients since the CMBD do not provide this information. We described the clinical form of disease and classified it as: a) pulmonary; b) meningeal; c) cutaneous; d) intestinal; e) adenitis; f) retinitis; g) pericarditis; h) disseminated; i) unspecified.

We also collected data from the National Statistics Institute (INE by its acronym in Spanish) of the Spanish population born in endemic countries and its evolution along the study period and any travels to endemic areas from the Tourist Studies institute (IET by its acronym in Spanish) [16,17].

The incidence rate (cases per 100,000 population-year) was calculated for the period 2002 to 2014 due to the lack of information from previous years. The population considered at risk were those living in Spain but born in endemic countries and people residing in Spain and travelling to endemic countries. We did not consider readmissions as new cases for this rate.

Categorical data were presented as absolute numbers and proportions, and continuous variables were expressed as mean and standard deviation, or medians and interquartile range when they did not present normal distribution. The χ2 test was used to compare categorical variables between groups. When comparing continuous variables between two groups the Student´s t test was used if they presented normal distribution, and Mann-Whitney U test if they did not. Also, we specified the two compared groups. Kruskal Wallis test was used when comparing continuous variables between more than two groups, since they did not present normal distribution. The Pearson correlation coefficient was used to evaluate the correlation between data. Results were considered statistically significant if the 2-tailed p value was <0.05. SPSS software for Windows (Version 19.0; SPSS Inc, Chicago, IL, USA) was used for statistical analyses.

### Ethics statement

This study used information from the CMBD. These data were totally anonymous, and researches had to request them directly to the MSSSI^13^. Procedures were performed in accordance with the ethical standards laid down in the Declaration of Helsinki as revised in 2013, and the study protocol was approved by the Ethical Review Board of the Vall d’Hebron University Hospital (Barcelona, Spain).

## Results

**Fig 1:**
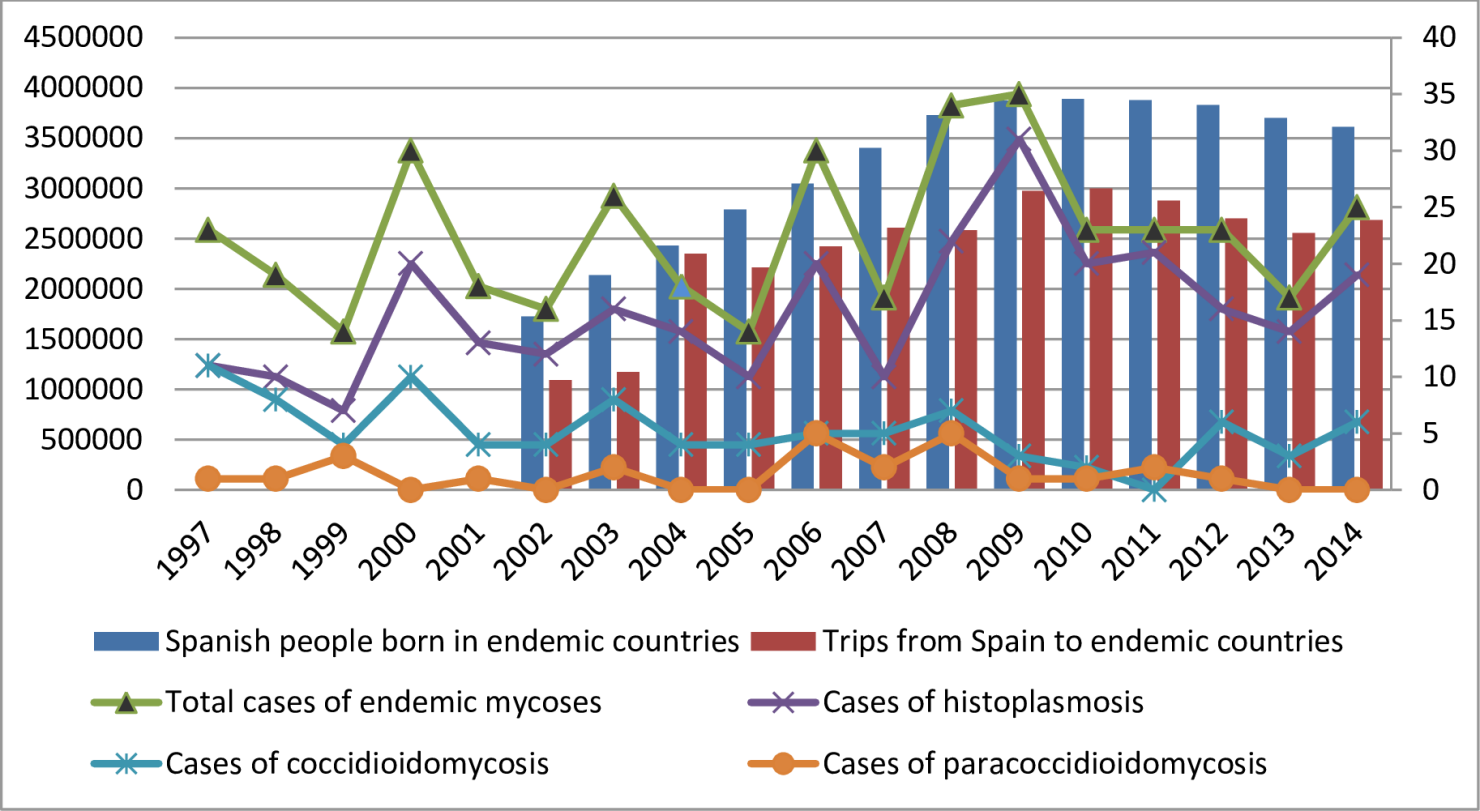
Evolution of cases of endemic mycoses and Spanish population at risk. *y1 axis: total number of people/trips in every year. *y2 axis: number of total cases in every year of the studied period, 1997–2014.

We received 461 entries with diagnosis of histoplasmosis, coccidioidomycosis or paracoccidioidomycosis from CMBD. After a manual review in which entries belonging to the same patient occurred after the initial diagnosis of endemic mycoses were not considered new cases of the disease, we found 405 new hospitalized cases, 286 (70.6%) of histoplasmosis, 94 (23.2%) of coccidioidomycosis and 25 (6.2%) of paracoccidioidomycosis. Fifty-six entries were considered readmissions. The evolution of cases throughout this period is presented in Fig 1.

### Clinical characteristics and burden of endemic mycoses in Spain

Overall, 266 of 405 cases were male (65.7%). The median age was 43 1(range 32–61.75) years old, and the mycosis infection was the main diagnosis in 204 cases (50.4%). The median hospitalization stay was 14 (range 6–31) days, 31 patients (7.7%) were readmitted at least once, and 54 (13.3%) deceased in the course of the disease.

Patients with histoplasmosis were significantly younger, had longer hospital stay and higher mortality rate compared with those with coccidioidomycosis. When compared to coccidioidomycosis, paracoccidioidomycosis was associated with longer hospital stay and patients were found to be younger. No differences in readmission rate were found. These data are presented in S1 Table.

### Histoplasmosis

There were 286 cases, of which 193 were male (67,5%). The median age of patients at admission was 37 (range 30–50) years old. A hundred eighty-eight patients (65,7%) were known to have an immunosuppressant condition, 151 (80.7%) of these had HIV infection. Furthermore, solid neoplasia was found in 14 patients (7.5%), organ transplant in 10 (5.3%), hematologic malignancy in 9 (4.8%), systemic autoimmune disease in 5 (2.7%), end stage chronic kidney disease in 5 (2.7%), hepatic cirrhosis in 3 (1.6%), and primary immunodeficiency in 2 (1.1%). Two concomitant immunosuppressant conditions were found in 12 of the 188 patients. The median hospital stay was 17 (range 8–35) days. In 124 cases (43,3%) histoplasmosis was the main diagnosis at discharge, 25 patients (8.7%) were readmitted at least once during the first year after discharge, and 44 patients (15.4%) deceased during the course of the disease.

Seventy-two patients (25.2%) presented isolated pulmonary disease, and 36 (12.6%) extrapulmonary disease. Within the extrapulmonary, 14 were diagnosed of meningeal histoplasmosis, 10 presented adenitis, 6 retinitis, 4 intestinal disease, 1 pericarditis, and 1 was classified as disseminated disease. No data were obtained from 168 patients (58.7%) and they were classified as unspecified disease. Moreover, 10 cases were caused by *Histoplasma capsulatum var*. *duboisii*, without specifying the clinical disease.

We compared clinical and epidemiological data from patients with histoplasmosis according to the competence of their immune system. Since HIV infection may play an important role in the burden of this disease HIV patients were analyzed separately from the immunosuppressed group. HIV patients were significantly younger and had longer hospital stay compared to other two groups, and had higher readmission rate when compared to immunocompetent individuals. Having an immunodeficiency was associated with higher in-hospital mortality when compared to immunocompetent patients. Immunocompetent patients presented more cases of extrapulmonary disease compared to other two groups. This analysis is presented in S2 Table.

The overall incidence rate from 2002 to 2014 ranged from 0.53 cases pero 100,000 population-year when considering just Spanish population born in endemic countries to 0.3 cases per 100,000 population-year when taking into account the total population at risk. The evolution of this rate is represented in S4 Table.

### Coccidioidomycosis

There were 94 cases, of which 57 were male (60.1%). The median age of patients at admission was 62.5 (range 48.75–72.25) years old. Twenty-three (24.5%) patients had a known immunosuppressant condition. Solid neoplasia was found in 13 patients (56.5%), followed by HIV infection in 3 (13%), systemic autoimmune disease was found in 2 (8.7%), hepatic cirrhosis in 2 (8.7%), end stage chronic kidney disease in 1 (4.3%), and hematologic malignancy in 1 (4.3%). The median hospitalization was 7 (3–15) days. In 59 cases (62.8%) coccidioidomycosis was the main diagnosis at discharge, 3 patients (3.2%) were readmitted during the first year after discharge, and 7 (7.4%) deceased during the course of the disease.

Forty-three patients (45.7%) presented isolated pulmonary disease, and 23 (19.3%) had extrapulmonary disease. Within them, 13 were diagnosed of meningitis, 7 presented cutaneous form, and 3 cases were classified as disseminated disease. No data about clinical presentation were obtained in 28 patients (29.8%) and they were classified as unspecified.

Since HIV infection was not the main immunosuppressive condition, we decided to compare only two groups in the statistical analysis, immunocompromised and immunocompetent patients. We only found that the immunocompromised group had a significantly higher readmission rate. This analysis is presented in S3 Table.

From 2002 to 2014, the overall incidence rate was 0.13 per 100,000 population-year when considering the total population at risk, and 0.14 cases per 100,000 population-year when taking into account just Spanish population born in endemic countries. The evolution of this rate is also represented in S4 Table.

### Paracoccidioidomycosis

There were 25 cases, of which 16 were male (64%). The median age of patients at admission was 47.5 (range 32.75–66.5) years old. Eight (32%) patients had known immunosuppressive condition. Solid neoplasia was found in 2 patients, HIV infection in 2, systemic autoimmune disease in 2, hematologic malignancy in 1, and terminal chronic kidney disease in 1 patient. The median hospitalization stay was 13.5 (4.25–26.75) days. In 21 cases (84%) paracoccidioidomycosis was the main diagnosis at discharge, 3 patients (12%) were readmitted during the first year after discharge, and 3 (12%) deceased during the course of the disease.

No data about clinical presentation could be obtained for paracoccidioidomycosis cases, due to the coding system used.

The groups for the statistical analysis were the same as in coccidioidomycosis, and we found no statistically significant differences in any studied variable. This analysis is presented in S3 Table.

From 2002 to 2014, the incidence rate of this disease was 0.03 per 100,000 population-year when considering the total population at risk, and 0.045 cases per 100,000 population-year when taking into account just Spanish population born in endemic countries. The evolution is represented in S4 Table.

### Spatial and temporal trends in Spain

**Fig 2:**
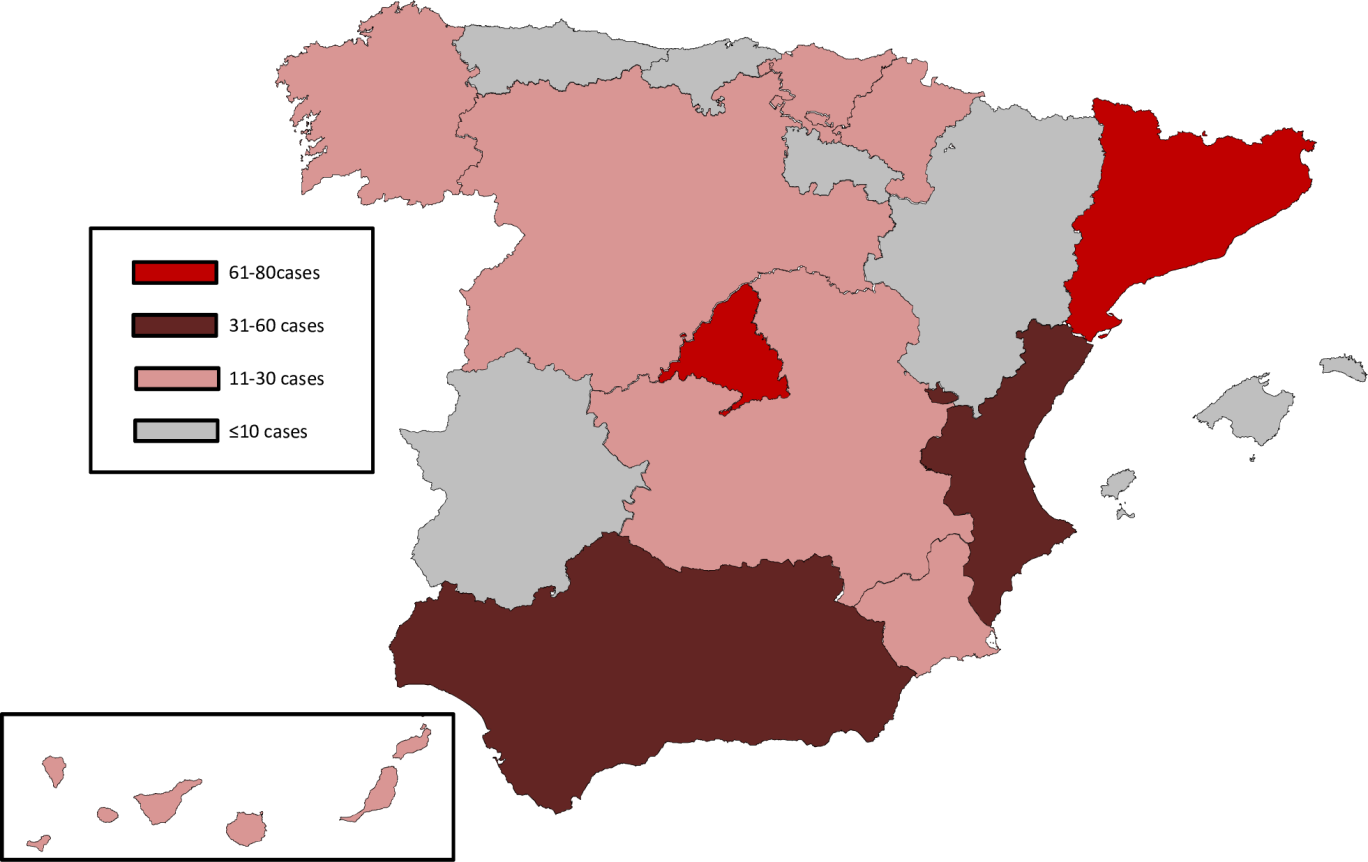
Distribution of cases of endemic mycoses according to the Autonomous Community to which the hospital belongs. The map was obtained from open access website www.d-maps.com, and it was modified with the software Inkscape.

The most affected autonomous communities were, in order of frequency: Madrid Community, with 92 cases (72 of histoplasmosis, 13 of coccidioidomycosis and 7 of paracoccidioidomycosis); Catalonia, with 74 cases (64 of histoplasmosis, 8 of coccidioidomycosis and 2 of paracoccidioidomycosis); Andalusia, with 44 cases (36 of histoplasmosis and 8 of coccidioidomycosis); and Valencian Community, with 42 cases in total (26 of histoplasmosis, 13 of coccidioidomycosis and 3 of paracoccidioidomycosis). These four communities are the most populated regions, with approximately 60% of total population of Spain [16]. They added up 252 of the 405 total cases (62.2%), but by January 1st 2015 they also beared 65.1% of population born in endemic countries: 844.286 lived in Catalonia, 747.803 in Madrid, 393.033 in Andalusia and 362.707 in Valencian Community [16]. The geographical distribution of cases in Spain is presented in Fig 2 and S4 Table. The Fig 2 represents the total cases of all together endemic mycoses in every Autonomous Community in Spain along the studied period.

In 2002, the first year when records from foreign population from the INE were available, there were 2,334,098 people in Spain who were born in foreign countries, and 1,484,652 of them were born in endemic countries. This situation increased gradually until 2010, when there were 3,887,935 people born in endemic countries. After that the numbers have progressively decreased, and at the end of the study period, 1st January 2015, there were 3,605,374 people [16].

In 2002 there were a total of 1.094.190 registered travels to endemic countries, and in 2010 the largest number was reached, with 2.999.730 travels. Since then, it also has suffered a progressive decline, until 2.684.096 travels in 2014 [17].

We found in the statistical analysis a weak correlation between the evolution of the number of cases of imported endemic mycoses and the migratory changes between 2002 and 2014 in Spain. We analyzed the correlation between the histoplasmosis cases and all mycoses cases separately, compared to population born in endemic countries and also to total population at risk. The highest correlation was found between histoplasmosis cases and total population born in endemic countries (Pearson correlation coefficient = 0.529). The calculation of this coefficient is represented in S5 Table.

## Discussion

Current demographic changes due to immigration and international movements result in an increase of imported diseases cases in non-endemic countries. We present in this study a reflection of this situation with imported endemic mycoses in Spain. Since only small series of these diseases have been reported and notification of these cases is not mandatory, we decided to use hospital records. During the total study period over 18 years, there were 286 hospitalizations for histoplasmosis, 94 for coccidioidomycosis and 25 for paracoccidioidomycosis. The last review was published in 2012, based in all cases reported in the literature since 1970. They found 128 cases of histoplasmosis and 21 cases of paracoccidioidomycosis^,^ and no data about coccidioidomycosis were obtained[1].

These infections have a high morbi-mortality, especially conditioned by the immune status of the individual, were HIV infection may play an important role. In respect to this fact, in the early HIV epidemic, histoplasmosis was diagnosed in 5 to 27% of patients with AIDS living in endemic areas, and more than 90% of them had disseminated disease, being considered as a defining AIDS illness [18]. In our study, more than a half of patients (52.8%) affected of histoplasmosis were infected by HIV, similar to other series described in endemic countries. The Colombian national survey described 434 cases of histoplasmosis between 1992–2008, and 70.5% of them were infected by HIV [19], and a Brazilian series of 73 patients found a HIV prevalence of 49% [20].

However, the immune status of the individual was not so important for coccidioidomycosis and paracoccidioidomycosis according to the results from our study, in which most of patients were immunocompetent, and HIV infection was not the main reason for immunodeficiency. In a Californian retrospective series of 223 cases of coccidioidomycosis, only one patient was infected by HIV [21]. Nevertheless, according to previous studies, immunosuppression could affect the clinical presentation of both diseases, by increasing the risk of extrapulmonary dissemination and mortality [22–24].

About the clinical characteristics related to these diseases, histoplasmosis was associated with longer hospitalization and mortality only compared to coccidioidomycosis. Patients with histoplasmosis were younger and had more frequently HIV infection than patients with other endemic mycoses.

As explained before, for histoplasmosis we compared three different patient groups according to their immune status. From our study, patients infected by HIV were younger and had longer hospital stay compared to the other two groups, and had higher proportion of readmissions compared to the non immunocompromised group. Both immunocompromised groups had higher mortality rate compared to immunocompetent patients, although it was higher in HIV patients, with 19.9% mortality. This rate is in concordance with other studies carried out in Latin America, that reported an approximate overall mortality of 30% in HIV patients with disseminated histoplasmosis [25]. However, this mortality rate was described only in AIDS patients without antiretroviral treatment (ART), and it was significantly lower in patients who were under ART [25]. In our study we were unable to know if patients were or not under ART, so we could not calculate the different mortality rates. The mortality of our immunocompetent patients was 7.1%, much lower than described in previous studies where mortality was 17%, however this study only considered cases of disseminated disease [26].

Reflecting the lesser role of immunosuppression in the other mycoses compared to histoplasmosis, we found no differences between the two analyzed groups for paracoccidioidomycosis, and for coccidioidomycosis we only found higher readmission rate in the immunocompromised group. They had also higher mortality, although it was not found to be significant. The overall mortality rate for coccidioidomycosis was 7.4%, higher than that found in previous literature that was around 3% one year after follow-up [27].

Focusing on the clinical pictures, it was difficult to assess the clinical spectrum since in more than half of the cases of histoplasmosis and around 30% of coccidioidomycosis were classified as unspecified, and no data were obtained for paracoccidioidomycosis. Pulmonary presentation was the most frequent. Thus, we decided to consider pulmonary and extrapulmonary as groups for the statistical analysis, and we found that immunocompetent patients had significantly more extrapulmonary histoplasmosis than immunocompromised patients. This does not concur with what is described in previous studies [28]. This is probably due to the lack of information about clinical presentations (67.5% of immunocompromised patients), and these results probably would change if the information was available.

We calculated the incidence rate of these diseases in Spain, and as expected they were much lower than those described in endemic countries. In endemic areas of United States, the estimated incidence of histoplasmosis in adults is 3.4 cases per 100,000 population-year, even higher in the Midwest of the country, with 6.1 cases per 100,000 population-year [29]. For coccidioidomycosis the estimated number of infections per year in the United States is approximately 150,000, and the incidence increased from 5.3 cases per 100,000 population in 1998 to 42.6 cases per 100,000 population in 2011 [30]. For paracoccidioidomycosis, an epidemiologic Brazilian study reported a mean incidence of 2.7 cases per 100,000 population-year, with a stable incidence between 1960 and 1999[8]. We calculated the incidence rate for every year, trying to reflect the trends of these diseases along the studied period. However, these rates were not expected to have great variations since the number of total cases was small.

Finally, we studied the possible correlation between the evolution of cases throughout the study period and the people at risk of having these infections. We found a positive correlation, but probably lower than expected. We studied separately histoplasmosis and all endemic mycoses cases, due to the larger number of cases of histoplasmosis compared to the other studied mycoses, and we studied also separately people living in Spain born in endemic countries and all people at risk, that means the sum of them and people who travel to endemic areas. We found the highest correlation between cases of histoplasmosis and the total population born in endemic countries. This reflects in some way, as expected, that the number of cases of histoplasmosis diagnosed in Spain has been increasing proportionally to the increase of immigration coming from endemic countries.

The main limitation of our study is the source of data collection. Since only the hospitalized and recorded cases of the disease are available, we did not obtain all cases diagnosed in Spain. We also were unable to obtain information about the diagnostic methods of the centers. Due to the coding system, we missed information about the clinical presentation of the cases, and for the assessment of the clinical evolution of the patients, we were only able to know if the patients were readmitted or deceased during the hospitalization, but we did not know if the mycosis was the main cause of this outcome and the long-term prognosis. Moreover, no data about treatment could be retrieved.

Even if we were able to obtain the total number of trips from Spain to endemic countries for every year, it is likely that most of these trips were made by people born in endemic countries who travelled to visit their relatives, so many of the patients in our study had both risk factors. This could be a bias when the incidence rates and the correlation coefficient are calculated, so we decided to calculate them in two ways: taking into account the total population at risk, and just considering Spanish population born in endemic countries (S4 Table and S5 Table). In addition, these diseases are probably underdiagnosed in non-endemic countries, since they are not initially suspected. Moreover, we were not able to evaluate the contribution of each endemic country to the cases of endemic mycoses in Spain because we did not obtain the nationality of the patients.

In conclusion, we presented a large series of cases of endemic mycoses in a non endemic country along the last years that reflect the increasing importance of these diseases due to demographic changes in our society. However, it is difficult to draw clear conclusions about the trends of these diseases in Spain due to the important limitations explained before. Despite these limitations, it covers all hospitalized cases diagnosed in Spain, and partially represents the clinical spectrum of these diseases, often underdiagnosed in non-endemic countries.

## Supporting information

S1 Table Clinical characteristics of hospitalized cases of imported endemic mycoses in Spain, 1997–2014. (DOCX)

S2 Table Clinical characteristics of hospitalized cases of histoplasmosis in Spain, 1997–2014. (DOCX)

S3 Table Clinical characteristics of hospitalized cases of other endemic mycoses in Spain, 1997–2014. (DOCX)

S4 Table Evolution of incidence rate of endemic mycoses in Spain, 2002–2014. (DOCX)

S5 Table Correlation of cases of endemic mycoses with migratory movements. (DOCX)
