# Supplementary material for: Imported endemic mycoses in Spain: Evolution of hospitalized cases, clinical characteristics and correlation with migratory movements, 1997-2014
Source: PLoS Negl Trop Dis. 2018 Feb 15;12(2):e0006245. doi: 10.1371/journal.pntd.0006245 (PMC5831632; doi:10.1371/journal.pntd.0006245)
Supplement: S1 Table — (DOCX) [file pntd.0006245.s001.docx]

|  | Total (n=405) | Histoplasmosis (H) (n=286) | Coccidioidomycosis (C) (n=94) | Paracoccidioidomycosis (P) (n=25) | P value |
| --- | --- | --- | --- | --- | --- |
| Male (n,%) | 266 (65.7%) | 193 (67.5%) | 57 (60.1%) | 16 (64%) | >0.05 |
| Age (median, interquartile range) | 43 (32-61.75) | 37 (30-50) | 62.5 (48.75-72.25) | 47.5 (32.75-66.5) | H Vs C=0.001  C Vs P=0.006 |
| Hospitalization stay (median, interquartile range) | 14 (6-31) | 17 (8-35) | 7 (3-15) | 13.5 (4.25-26.75) | H Vs C=0.001  C Vs P=0.026 |
| Patients readmitted (n,%) | 31 (7.7%) | 25 (8.7%) | 3 (3.2%) | 3 (12%) | >0.05 |
| Deaths (n,%) | 54 (13.3%) | 44 (15.4%) | 7 (7.4%) | 3 (12%) | H Vs C=0.048 |
| Principal diagnosis (n,%) | 204 (50.4%) | 124 (43.3%) | 59 (62.8%) | 21 (84%) | <0.05 |
