# Supplementary material for: Imported endemic mycoses in Spain: Evolution of hospitalized cases, clinical characteristics and correlation with migratory movements, 1997-2014
Source: PLoS Negl Trop Dis. 2018 Feb 15;12(2):e0006245. doi: 10.1371/journal.pntd.0006245 (PMC5831632; doi:10.1371/journal.pntd.0006245)
Supplement: S2 Table — (DOCX) [file pntd.0006245.s002.docx]

| Histoplasmosis  (n=286) | HIV patients^1^ (n=151) | Immunocompromised^2^ non HIV (n=37) | Non immunocompromised^3^ (n=98) | P value |
| --- | --- | --- | --- | --- |
| Male (n,%) | 100 (66.2%) | 27 (73%) | 67 (68.4%) | >0.05 |
| Age (median, interquartile range) | 35 (30-42) | 65 (49-73) | 38.5 (28-59) | <0.05 |
| Hospitalization stay (median, interquartile range) | 26 (13-39) | 10 (6-21.5) | 12.5 (4-23) | 1 Vs 2=0.001  1 Vs 3=0.001 |
| Extrapulmonary form (n,%)* | 15 (9.9%) | 2 (5.4%) | 19 (19.3%) | 1 Vs 3=0.018  2 Vs 3=0.046 |
| Patients readmitted (n,%) | 17 (11.2%) | 4 (10.8%) | 4 (4.1%) | 1 Vs 3=0.048 |
| Deaths (n,%) | 30 (19.9%) | 7 (18.9%) | 7 (7.1%) | 1 Vs 3=0.005  2 Vs 3=0.044 |
| Principal diagnosis (n,%) | 22 (14.6%) | 20 (54%) | 82 (83.7%) | <0.05 |

*168 cases (58.7%) were classificated as “unspecified”, and 10 (3.5%) were produced by H. Capsulatum var. Duboisii
