# Supplementary material for: Imported endemic mycoses in Spain: Evolution of hospitalized cases, clinical characteristics and correlation with migratory movements, 1997-2014
Source: PLoS Negl Trop Dis. 2018 Feb 15;12(2):e0006245. doi: 10.1371/journal.pntd.0006245 (PMC5831632; doi:10.1371/journal.pntd.0006245)
Supplement: S3 Table — (DOCX) [file pntd.0006245.s003.docx]

| Other mycoses (n=119)  Coccidioidomycosis, n=94  Paracoccidioidomycosis, n= 25 | Immunocompromised 23 (24.5%)  8 (32%) | Non immunocompromised 71 (75.5%)  17 (68%) | P value |
| --- | --- | --- | --- |
| Male (n,%) |  |  |  |
| Coccidioidomycosis | 14 (60.1%) | 43 (60.1%) | 0.999 |
| Paracoccidioidomycosis | 5 (62.5%) | 11 (64.7%) | 0.914 |
| Age (mean, standard deviation) |  |  |  |
| Coccidioidomycosis | 57.3 (16) | 61 (18.3) | 0.383 |
| Paracoccidioidomycosis | 49.1 (20.6) | 47.1 (18.7) | 0.805 |
| Hospitalization stay (median, interquartile range) |  |  |  |
| Coccidioidomycosis | 9 (6-16) | 7 (3-14) | 0.083 |
| Paracoccidioidomycosis | 9 (4.5-19.25) | 17 (4.5-28) | 0.44 |
| Extrapulmonary form (n,%) |  |  |  |
| Coccidioidomycosis* | 8 (25.8%) | 15 (17%) | 0.351 |
| Paracoccidioidomycosis** | - | - | - |
| Patients readmitted (n,%) |  |  |  |
| Coccidioidomycosis | 3 (13%) | 0 | 0.002 |
| Paracoccidioidomycosis | 0 | 3 (17.7%) | 0.204 |
| Deaths (n,%) |  |  |  |
| Coccidioidomycosis | 3 (13%) | 4 (5.7%) | 0.247 |
| Paracoccidioidomycosis | 0 | 3 (17.7%) | 0.204 |
| Principal diagnosis (n,%) |  |  |  |
| Coccidioidomycosis | 8 (34.8%) | 51 (71.8%) | 0.001 |
| Paracoccidioidomycosis | 5 (62.5%) | 16 (94.1%) | 0.044 |

*28 cases (29.8%) of were classificated as “unspecified”; **No data about clinical forms were obtained
