# Supplementary material for: Imported endemic mycoses in Spain: Evolution of hospitalized cases, clinical characteristics and correlation with migratory movements, 1997-2014
Source: PLoS Negl Trop Dis. 2018 Feb 15;12(2):e0006245. doi: 10.1371/journal.pntd.0006245 (PMC5831632; doi:10.1371/journal.pntd.0006245)
Supplement: S4 Table — (DOCX) [file pntd.0006245.s004.docx]

| Year | Spanish population born in endemic countries | Trips from Spain to endemic countries | Total population at risk | Endemic mycosis | Cases | Incidence rate 1* | Incidence rate 2** |
| --- | --- | --- | --- | --- | --- | --- | --- |
| 1997 |  |  |  | Histoplasmosis  Coccidioidomycosis  Paracoccidioidomycosis | 11  11  1 |  |  |
| 1998 |  |  |  | Histoplasmosis  Coccidioidomycosis  Paracoccidioidomycosis | 10  8  1 |  |  |
| 1999 |  |  |  | Histoplasmosis  Coccidioidomycosis  Paracoccidioidomycosis | 7  4  3 |  |  |
| 2000 |  |  |  | Histoplasmosis  Coccidioidomycosis  Paracoccidioidomycosis | 20  10  0 |  |  |
| 2001 |  |  |  | Histoplasmosis  Coccidioidomycosis  Paracoccidioidomycosis | 13  4  1 |  |  |
| 2002 | 1,725,672 | 1,094,190 | 2,819,862 | Histoplasmosis  Coccidioidomycosis  Paracoccidioidomycosis | 12  4  0 | 0.42  0.14  0 | 0.69  0.23  0 |
| 2003 | 2,138,436 | 1,173,754 | 3,312,190 | Histoplasmosis  Coccidioidomycosis  Paracoccidioidomycosis | 16  8  2 | 0.48  0.24  0.06 | 0.75  0.37  0.09 |
| 2004 | 2,430,190 | 2,351,806 | 4,781,996 | Histoplasmosis  Coccidioidomycosis  Paracoccidioidomycosis | 14  4  0 | 0.29  0.08  0 | 0.58  0.16  0 |
| 2005 | 2,788,335 | 2,213,998 | 5,002,333 | Histoplasmosis  Coccidioidomycosis  Paracoccidioidomycosis | 10  4  0 | 0.2  0.08  0 | 0.36  0.14  0 |
| 2006 | 3,048,459 | 2,422,591 | 5,471,050 | Histoplasmosis  Coccidioidomycosis  Paracoccidioidomycosis | 20  5  5 | 0.36  0.09  0.09 | 0.66  0.16  0.16 |
| 2007 | 3,402,992 | 2,609,435 | 6,012,427 | Histoplasmosis  Coccidioidomycosis  Paracoccidioidomycosis | 10  5  2 | 0.17  0.08  0.03 | 0.29  0.15  0.06 |
| 2008 | 3,727,552 | 2,582,935 | 6,310,487 | Histoplasmosis  Coccidioidomycosis  Paracoccidioidomycosis | 22  7  5 | 0.35  0.11  0.08 | 0.59  0.19  0.13 |
| 2009 | 3,875,341 | 2,973,475 | 6,848,816 | Histoplasmosis  Coccidioidomycosis  Paracoccidioidomycosis | 31  3  1 | 0.45  0.04  0.01 | 0.8  0.08  0.03 |
| 2010 | 3,887,935 | 2,999,730 | 6,887,665 | Histoplasmosis  Coccidioidomycosis  Paracoccidioidomycosis | 20  2  1 | 0.29  0.03  0.01 | 0.51  0.05  0.03 |
| 2011 | 3,876,695 | 2,879,538 | 6,756,233 | Histoplasmosis  Coccidioidomycosis  Paracoccidioidomycosis | 21  0  2 | 0.31  0  0.03 | 0.54  0  0.05 |
| 2012 | 3,830,424 | 2,702,906 | 6,533,330 | Histoplasmosis  Coccidioidomycosis  Paracoccidioidomycosis | 16  6  1 | 0.24  0.09  0.02 | 0.42  0.16  0.03 |
| 2013 | 3,699,391 | 2,556,918 | 6,256,309 | Histoplasmosis  Coccidioidomycosis  Paracoccidioidomycosis | 14  3  0 | 0.22  0.05  0 | 0.38  0.08  0 |
| 2014 | 3,611,815 | 2,684,096 | 6,295,911 | Histoplasmosis  Coccidioidomycosis  Paracoccidioidomycosis | 19  6  0 | 0.3  0.1  0 | 0.52  0.17  0 |

*Calculated taking into account “total population at risk” **Calculated taking into account just “Spanish population born in endemic countries”
