# Supplementary material for: Imported endemic mycoses in Spain: Evolution of hospitalized cases, clinical characteristics and correlation with migratory movements, 1997-2014
Source: PLoS Negl Trop Dis. 2018 Feb 15;12(2):e0006245. doi: 10.1371/journal.pntd.0006245 (PMC5831632; doi:10.1371/journal.pntd.0006245)
Supplement: S5 Table — (DOCX) [file pntd.0006245.s005.docx]

| Pearson coefficient | Histoplasmosis cases | Total endemic mycoses cases |
| --- | --- | --- |
| Spanish population born in endemic countries | 0.529 | 0.398 |
| Total population at risk | 0.517 | 0.360 |
